# Impact of suspending minimum volume requirements for knee arthroplasty on hospitals in Germany: an uncontrolled before–after study

**DOI:** 10.1186/s12913-020-05957-1

**Published:** 2020-12-01

**Authors:** Werner de Cruppé, Annette Ortwein, Rike Antje Kraska, Max Geraedts

**Affiliations:** 1 Institute for Health Services Research and Clinical Epidemiology, Philipps-Universität Marburg, Karl-von-Frisch-Strasse 4, 35043 Marburg, Germany; 2 Institute for Health Systems Research, School of Medicin, Faculty of Health, Witten/Herdecke University, Alfred-Herrhausen-Strasse 50, 58448 Witten, Germany

**Keywords:** Minimum volume requirement, Minimum caseload, Volume-outcome relationship, Hospital, Inpatients, Uncontrolled before–after study, Total knee arthroplasty, Germany

## Abstract

**Background:**

In 2004, the Federal Joint Committee, supreme decision-making body in German healthcare, introduced minimum volume requirements (MVRQs) as a quality instrument. Since then, MVRQs were implemented for seven hospital procedures. This study evaluates the effect of a system-wide intermission of MVRQ for total knee arthroplasty (TKA), demanding 50 annual cases per hospital.

**Methods:**

An uncontrolled before–after study based on federal-level data including the number of hospitals performing TKA, and TKA cases from the external hospital quality assurance programme in Germany (2004–2017). Bi- and multivariate analyses based on hospital-level secondary data of TKA cases and TKA quality indicators extracted from hospital quality reports in Germany (2006–2014).

**Results:**

The number of TKAs performed in Germany decreased by 11% after suspending the TKA-MVRQ in 2011, and rose by 13% after its reintroduction in 2015. The number of hospitals with less than 50 cases rose from 10 to 25% and their case share from 2 to 5.5% during suspension. Change in hospital volume after the suspension of TKA-MVRQ was not associated with hospital size, ownership, or region. All four evaluable quality indicators increased significantly in the year after their first public reporting. Compared to hospitals meeting the TKA-MVRQ, three indicators show slight but statistically significant better quality in hospitals below the TKA-MVRQ.

**Conclusions:**

In Germany, TKA-MVRQs seem to induce in-hospital caseload adjustments rather than foster regional inter-hospital case transfers as intended.

## Background

Minimum volume requirements (MVRQs) are an internationally adopted quality instrument. In his pioneering publication in 1979, Luft analysed “whether there is a relation between a hospital’s surgical volume and its surgical mortality” [1]. He found an inverse relation of procedure volume and mortality that has been further investigated and frequently confirmed for numerous procedures in many studies and reviews [2]. Regionalisation of the respective treatments should, therefore, have a positive influence on treatment quality, and patients should be redirected from low volume hospitals to high volume hospitals [1]. The relation between procedure volume and outcome quality has been transformed into a health policy instrument by requiring minimum case numbers per hospital or surgeon per year. Thus, minimum volume requirements for hospitals are installed to improve treatment results for patients and to redistribute hospital caseloads. These two steering effects of minimum volume requirements have been used either by authorities as a universal requirement in public or social security-based health care systems [3–8], or as initiatives in privately organized health care systems [9, 10].

In Germany, MVRQs are implemented for seven hospital procedures as of 2020: Complex oesophageal interventions, complex pancreatic interventions, kidney transplantation, liver transplantation, stem cell transplantation, total knee arthroplasty, and infants with birth weight < 1250 g. Table 1 gives details on their introduction and minimum volumes [11, 12]. A further two procedures, for lung and breast cancer, are currently under debate. MVRQs were introduced into health policy as part of the Social Act V in 2002. It is assigned to the highest decision-making body of the joint self-government in the German health care system, the Federal Joint Committee (G-BA), in order to decide on a minimum volume regulation (MVRG) with defined procedures, thresholds, exemptions and penalties. The aims of the MVRG are stated in §2 [13]: 1. To ensure an adequate quality of care and the continuous improvement of the level of care. 2. The application of the minimum volumes established pursuant to this agreement shall neither jeopardise adequate access to care nor aggravate existing undersupply. 3. The minimum volume regulation must not contradict Advanced Training Regulations in their current version.

**Table 1:** Minimum volumes and requirements per hospital and year in Germany (table based on [11])

Type of intervention	2004–2005	2006–2009	2010	2011–2014	since 2015
Complex oesophageal interventions	5	10	10	10	10
Complex pancreatic interventions	5	10	10	10	10
Kidney transplantation	10	20	20	20	20
Liver transplantation	20	25	25	25	25
Stem cell transplantation	12	25	25	25	25
Total knee arthroplasty	–	50	50	suspended	50
Infants with birth weight < 1250 g	–	–	14	30 planned, 14 continued	14

This MVRG entered into force as from January 2004 after being approved in December 2003, thus granting hospitals a rather short timespan to implement the new regulations. From a health services research perspective, it has been unfeasible to collect appropriate baseline data on care delivery and quality status of minimum volume procedures on the hospital level before the regulation came into force, impeding the comparison of data from before and after this intervention. Hence, the first policy evaluation published in 2008 analysing the initial 3 years of MVRG was limited to the number of performing hospitals and their caseloads under MVRG [14–16], and the description of possible regionalisation effects if all hospitals were to comply with MVRQs [17–19]. However, in the following years, further studies on care delivery for minimum volume procedures found no evidence that Germany experienced regionalisation effects, especially regarding three non-transplantation procedures, i.e. complex oesophageal and pancreatic interventions, and TKA [20]. It remains unclear to what extent the initial introduction of MVRG in 2004 affected care delivery on the hospital level. Although all hospitals in Germany underlie an extensive external quality assurance programme since 1996, the main argument for introducing a MVRG is still difficult to evaluate, i.e. how MVRGs reduce potential harm and death on patients undergoing procedures in low volume hospitals [21]. In 2016, the external quality assurance programme covered 24 fields of intervention and collected data on 238 quality indicators, of which 216 were reported publicly in the hospitals’ compulsory annual quality report. The reported indicators are available for scientific use [22–24]. Unfortunately, only a few of the external quality assurance quality indicators correspond to the minimum volume procedures and even less have to be published. Furthermore, their utilization is limited by numerous changes in definition, impeding longitudinal comparison. Therefore, most quality evaluations of the MVRG in Germany focus on in-hospital mortality, as this is the only constantly available quality indicator of the hospital discharge dataset reported to the Laender and federal statistical office. Studies using these data have shown a predominantly positive relationship between higher caseloads and lower in-hospital mortality rates, considering numerous adjustment factors, for procedures under MVRG [21, 25] as well as for other procedures [26–30].

Introducing MVRQs for TKA in Germany has been under discussion regarding expected effects on mortality and complication rates [31–33], an appropriate procedure threshold [34–36], and regionalisation [32, 37, 38]. TKA-MVRQ requiring 50 procedures per hospital annually as performed by less than 1100 hospitals in Germany, came under criticism and gave reasons for a legal dispute up to the Federal Social Court between 2009 and 2014. This lawsuit involved the freedom of medical treatment for a hospital and its respective surgeons by delivering TKA, the reimbursement of its costs by the sickness fund, and the evidence for the threshold of 50 cases per year. The TKA-MVRQ was suspended from 10/2011 to 12/2014 and reintroduced in 2015 with the same, henceforth adjudicated threshold of 50 procedures per year per hospital. It is important to note that the Federal Joint Committee’s decision to suspend TKA-MVRQs came into force on October 19th in 2011, thus making 2011 an ambiguous year; for although TKA-MVRQ were suspended retrospectively for the entire year 2011, the hospitals acted on the assumption of unaltered MVRQs until October 2011. Therefore, part of the data collected in this year depicts MVRQ in force. Still, this legal dispute is understood to be an – unplanned and uncontrolled - intervention in the delivery of TKA-procedures in all German hospitals. Thus, it offers a chance to analyse the impact of introducing and suspending MVRQs in the German health care system as an uncontrolled before-after-study.

Unlike hip arthroplasty, TKA is not among the operations Luft questioned to be regionalised [1]. It was not before 1997 that data on mortality after TKA were published for different hospital volumes and distinctly separated from hip arthroplasty [39], finding a combined in-house mortality rate of 0.25%, varying between low-volume (< 25 cases, 0.35%), medium-volume (25–199, 0.27%), and high-volume hospitals (> 199, 0.22%). A first review of the effect of TKA hospital volume on mortality and complication rates from 2004 reports on 13 studies and sums up data of five studies in a meta-analysis of TKA performed between 1985 and 1999. The study found consistent but very small effects on mortality rates (in-hospital mortality varying between 0.2 and 0.5%) and inconsistent complication rates between different hospital volumes [40]. A more recent review published in 2010 includes eleven studies on primary TKA performed until 2005 [41], finding that only the comparison between the highest and lowest volume category was connected significantly to a higher complication rate and three out of six studies (partially) confirmed the inverse connection of hospital volume and mortality. According to a review on procedure data from 1992 up to 2007 [42], surgeon volume and primary TKA were not associated with mortality, survivorship, and thromboembolic events but significantly inversely related to infections, transfusion rates, and patient reported outcomes. As with other procedures studied for volume effects, the volume thresholds are disparate among TKA studies, very low and low varying between 1 and 100 cases, medium between 20 and 200, and high and very high ranging from 40 to more than 500 [41, 42]. Since short-term complication and mortality rates generally show lower values than other surgical procedures, recent studies on TKA focus on revision rates associated with volume [43–45]. Reviewing risk factors for revision of TKA, Jasper et al. find 10-year survival rates in six studies of 89.5 to 98.6% [46]. An increased risk was associated with demographic factors such as age, surgical factors, e.g. implant alignment, and low volume hospitals.

The first evaluation of quality for TKA performed in Germany was carried out for two short-term outcomes, i.e. postoperative wound infection and wound hematoma / secondary haemorrhage, using external quality assurance data of all German hospitals of the years 2004 to 2006. This revealed a reduction of postoperative wound infection by 22.5%, and half of this improvement could be attributed to MVRQs. The improvement of wound hematoma / secondary haemorrhage could not be traced back to MVRQs [47]. A study on surgical site infection analysed the infection rate of 71 hospitals voluntarily participating in the German nosocomial infections surveillance system on TKA from 2003 up to 2008. It showed a significantly higher infection rate for hospitals not meeting the MVRQ of 50 TKA with 1.81% compared to those with 50 to 100 TKA (0.88%) and hospitals with more than 100 TKA (0.79%) [48]. Recent analyses of in-hospital mortality using the complete dataset of all inpatients in Germany of the years 2006–2013 with 1.1 million TKA showed adjusted mortality rates of 0.18% for hospitals below TKA-MVRQ and 0.13% for hospitals above. The adjusted OR of 0.79 (95% CI 0.55–0.90) indicates a significantly lower mortality rate in hospitals above TKA-MVRQ [25]. These results were confirmed with the complete German dataset from 2009 to 2014 [21] calculating in-hospital mortality rates with case volume categorized in quintiles.

The literature review on minimum volume for TKA to date reveals a lack of studies regarding the impact of introducing and suspending TKA MVRQ in a health care system. Therefore, the study at hand focuses on the effects of MVRQ for TKA in the German health care system with TKA-MVRQ in force from 2006 to 2010, the uniform and system-wide suspension from 2011 to 2014, and its reintroduction in 2015, considered as an uncontrolled intervention. The aim of this study is to analyse the impact on the health care delivery process by counting annual numbers of performing hospitals and their caseloads on an aggregated federal level as well as on the hospital level. Subsequently, the findings are combined with quality of care as measured by TKA quality indicators (TKA-QI) of the official German external quality assurance programme, published in hospital quality reports.

## Methods

Our study design is an uncontrolled before–after study; the target population is all hospitals in Germany performing TKA. The intervention consists of the Federal Joint Committee’s decision to suspend TKA-MVRQ of 50 annual cases per hospital for all TKA-performing hospitals in Germany in 2011, lasting until the end of 2014, and its reintroduction from 2015 on. It is uncontrolled, since the intervention is uniform and system-wide, impeding a control-group design.

Our study is based on secondary data from two sources, i.e. external quality assurance data and quality reports of German hospitals. All hospitals performing TKA report external quality assurance data for each patient undergoing TKA in a structured data collection, validated by the external quality assurance programme. The Institute for Quality Assurance and Transparency in Healthcare controls, evaluates, and publishes the data aggregated on the federal level, offering annual statistics of all TKA cases and TKA-performing hospitals. Hence, this dataset covers the time before TKA-MVRQ from 2004 to 2005, with TKA-MVRQ in force from 2006 to 2011, its suspension until 2014, and its reintroduction from 2015 up to 2017. All data are publicly available. We extracted the supplied dataset on TKA cases and hospital numbers for each year as reported in the basic statistic. These data serve to answer the first study aim on an aggregated federal level. It is to note that, in all years under investigation, all shown external quality assurance TKA data refer uniformly to TKA cases, to allow a consistent comparison. Thus, we refer to the majority of knee replacements with the total knee joint being replaced, including bicondylar endoprosthesis, cemented or uncemented, constrained or unconstrained, with or without the replacement of the patellar, or bicompartmental knee arthroplasty. Neither partial or unicompartmental knee arthroplasties, nor revisions of knee replacements are included in the data. Unicompartmental knee arthroplasty procedures are part of the external quality assurance programme since 2015 and revisions of knee replacements since 2003. To indicate the proportion between the procedures, the external quality assurance programme reports 134,224 TKAs (78%), 19,814 unicompartmental knee arthroplasties (12%), and 17,677 revisions (10%) for Germany in 2015.

Quality reports of German hospitals are the second source, providing information on hospital size, location, and type of ownership in the years available (2006, 2008, 2010, 2012, 2013, 2014). They include external quality assurance data on TKA-QI on the hospital level, limited to QIs compulsory for public reporting. The quality reports are mandatory structured report cards with detailed reporting instructions defined by the Federal Joint Committee, completed by each hospital site, and forwarded to a public collecting agency. Having added quality indicator data of the external quality assurance programme compulsory for publication for each hospital, the agency publishes all reports online. Until 2012, the reports were due every second year, since then they are published annually. The Federal Joint Committee provides the dataset free of charge, upon written request and with contractually agreed terms of use. The reports from 2006, 2008 and 2010 depict the situation with TKA-MVRQ in force, while those from 2012, 2013, and 2014 are data during the intervention, i.e. with TKA-MVRQ suspended. For the year 2011, no quality reports were due. Our study population under investigation is all hospitals reporting a TKA in at least 1 year, and available quality reports for all years covered by the dataset. All six quality reports of each hospital are linked, allowing longitudinal analyses on the hospital level. The data linkage is well established and is subject of another publication [49]. All hospitals with their quality reports available and linked are further on referred to as ‘study population’. If quality reports could not be associated with a single hospital or a hospital site, they were excluded from the final dataset.

We defined the ‘annual TKA case volume per hospital’ as the department procedure volume of TKA as given in the second section of the quality report for each hospital. Hospitals with one to three cases are obliged to report “caseload: < 4” due to data protection regulations of the Federal Joint Commission following German data protection laws to preserve the patients’ anonymity; we set the caseload of these 0.9% of all hospitals to two cases per hospital for calculation purposes.

Data on all ten TKA-QIs mandatory for publishing in the quality reports were considered as the dependent variables to study hospital quality. All TKA-QIs are listed in Table 2 including detailed information on data availability. The statistical analysis of the TKA-QIs was limited due to different reasons. For most years, only a subset of the TKA-QIs was available, all ten are solely available for 2013 and 2014. Furthermore, three QIs with very low case numbers per hospital (postoperative wound infection, reoperation due to complications, in-hospital mortality) and their respective ratios of observed vs. expected rates could not be analysed because of massive underreporting due to data protection regulations prohibiting the publication of data on hospitals with less than four cases. Finally, only four TKA-QIs could be analysed, as their definitions were consistent over the years under study. These indicators are: TKA meets the indication criteria, perioperative antibiotic-prophylaxis, postoperative mobility (neutral zero method), and postoperative mobility of at least 0/0/90 (neutral zero method). The optimum value for these QIs is uniformly defined as 100% to facilitate comparability between all four QIs. Analyses beyond 2014 are impeded by major changes in the TKA-QI-set, their definitions, and availability due to their official suspension by the Federal Joint Committee for public reporting on the hospital level for 2015 and following years.

**Table 2:** Available years for total knee arthroplasty (TKA) quality indicators in quality reports (table based on [22])

TKA Quality Indicator	2006	2008	2010	2012	2013	2014
^a^TKA meets indication criteria	No	No	Yes	Yes	Yes	Yes
^a^Perioperative antibiotic prophylaxis applied	No	No	No	Yes	Yes	Yes
^a^Postoperative mobility (neutral zero method) measured	No	No	Yes	Yes	Yes	Yes
^a^Postoperative mobility of at least 0/0/90 (neutral zero method)	No	No	Yes	Yes	Yes	Yes
Postoperative wound infection	Yes	Yes	No	No	Yes	Yes
Ratio of observed to expected rate (o/e) of postoperative wound infections	No	No	No	No	Yes	Yes
Reoperation due to complications	Yes	Yes	Yes	No	Yes	Yes
Ratio of observed to expected rate (o/e) of reoperations due to complications	No	No	No	No	Yes	Yes
In-hospital mortality	No	No	Yes	Yes	Yes	Yes
Ratio of observed to expected rate (o/e) of in-hospital mortality	No	No	No	Yes	Yes	Yes

^a^Quality indicator data availability **not** limited by data protection regulations and with consistent definition

To determine the MVRG effects, frequencies of performing hospitals and their case numbers are presented per year from the external quality assurance data and the quality reports. Comparing both data sources also validates the study population of hospitals providing longitudinal data from the quality reports in contrast to all TKA data of the external quality assurance programme, aggregated on the federal level. We applied a multifactorial analysis of variance with repeated measures to analyse the influence of the independent variables time, hospital size categories (<=100 beds, > 100 < = 200, > 200 < = 300, > 300 < = 500, > 500 beds), geographical region in Germany (north-west, south, east), type of ownership (charitable (non-profit), public (non-profit), private (for-profit)), and interactions of time with the latter three variables, on hospitals performing less than the minimum 50 cases per year or 50 or more cases per year before and after suspending the TKA-MVRQ. The chosen bed size categories follow the categorisation established by the German Federal Statistical Office and approximate an even distribution of hospitals in the study population [50]. Table 3 shows the distribution of hospitals in the study population compared to all hospitals in Germany in 2013 by hospital size categories, region and type of ownership. The Greenhouse–Geisser adjustment corrected for violations of sphericity. We performed Bonferroni-adjusted post-hoc-tests to determine significant differences between group means. The non-parametric Friedman test including Bonferroni-corrected pairwise comparisons of hospitals TKA case numbers between the 6 years under observation was applied to confirm the findings.

**Table 3:** Hospitals of the study population compared to all hospitals in Germany [50]

	Germany 2013		Hospitals of the study population	
Beds	N	%	N	%
<=100	693	34,7	87	8,2
> 100 < = 200	432	21,6	245	23,2
> 200 < = 300	273	13,7	217	20,6
> 300 < = 500	337	16,9	274	26,0
> 500	261	13,1	232	22,0
Sum	1996	100	1055	100
**Geographical region in Germany**				
North-west	728	36,5	413	39,1
South	922	46,2	443	42,0
East	346	17,3	199	18,9
	1996	100	1055	100,0
**Type of ownership**				
Public (non-profit)	596	29,9	416	39,4
Charitable (non-profit)	706	35,4	426	40,4
Private (for-profit)	694	34,8	213	20,2
	1996	100	1055	100,0

The data for the second study aim, to detect effects of case volume on quality of care, was analysed comparing TKA-QI mean differences of hospitals with less than 50 cases with hospitals performing 50 or more annual cases (TKA-MVRQ value), independently for each QI and each year (unpaired sample). Mann-Whitney-U-tests were used for analyses since all four TKA-QIs are not normally distributed. The common null hypothesis for all four tested quality indicators is: Hospitals complying with the MVRQ for TKA (with 50 and more TKA cases per year) achieve no different outcome in quality indicator values than hospitals not complying with the TKA MVRQ. Subsequently, the influence of suspending the MVRQ on these four TKA-QIs was analysed using a multifactorial MANOVA with a paired sample (four measurement years for each hospital and QI) followed by Bonferroni-adjusted post-hoc-tests. All analyses were conducted using the statistics programme SPSS 23.

## Results

### Study aim one

**Fig. 1:**
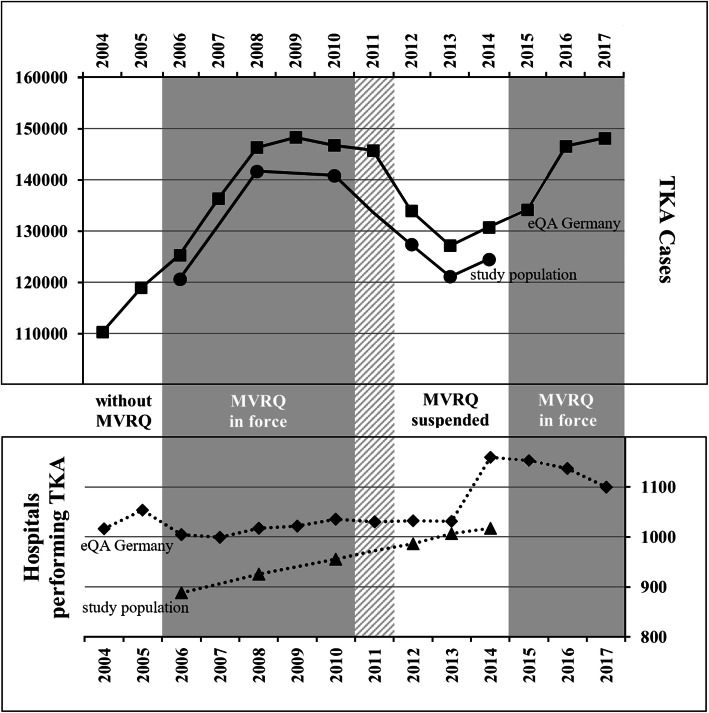
Number of total knee arthroplasty cases and hospitals performing TKA per year. TKA = total knee arthroplasty; MVRQ = minimum volume requirements; eQA = external hospital quality assurance programme

Aggregated external quality assurance data on the federal level document that the caseload increased by one third after MVRQ came into force, rising from 110,349 cases before the first MVRQ for TKA in 2004 to 146,318 in 2008 with the TKA-MVRQ in force from 2006 on (Table 4 and Fig. 1). The caseload then remained stable until 2011. After the TKA-MVRQ was suspended, the caseload fell by 11%, resulting in 127,192 cases in 2013 and, consequently, rose by 13% when the TKA-MVRQ was reintroduced in 2015, culminating in 148,160 cases in 2017. The number of TKA-performing hospitals remained rather constant over time at a level of 1030, until in 2014 it increased by 130 reported hospital sites, decreasing again by 60 hospitals until 2017.

**Table 4:** Number of total knee arthroplasty (TKA) cases and hospitals performing TKA per year in the study population compared to data from the external hospital quality assurance programme (eQA) [22]

	Cases: study population	Cases: eQA	Cases: difference between data sets	% Cases: difference between data sets	Hospitals: study population	Hospitals: eQA	Hospitals: difference between data sets	% Hospitals: difference between data sets
2004		110,349				1016		
2005		118,967				1054		
**2006**	**120,700**	**125,394**	**4694**	**3.7**	**888**	**1005**	**117**	**11.6**
2007		136,379				999		
**2008**	**141,714**	**146,318**	**4604**	**3.1**	**926**	**1017**	**91**	**8.9**
2009		148,298				1022		
**2010**	**140,866**	**146,747**	**5881**	**4.0**	**956**	**1036**	**80**	**7.7**
2011		145,750				1030		
**2012**	**127,404**	**133,948**	**6544**	**4.9**	**987**	**1033**	**46**	**4.5**
**2013**	**121,134**	**127,192**	**6058**	**4.8**	**1007**	**1031**	**24**	**2.3**
**2014**	**124,544**	**130,804**	**6260**	**4.8**	**1017**	**1160**	**143**	**12.3**
2015		134,224				1153		
2016		146,615				1137		
2017		148,160				1100

Our study population for longitudinal analyses on the hospital level consists of 1055 hospitals. The number of TKA-performing hospitals increased constantly from 888 in 2006 to 1017 in 2014; meanwhile, the total annual TKA case volume rose from 120,700 in 2006 to 140,866 in 2010, dropped to 127,404 in 2012 and remained on 124,544 in 2014 (Table 5). Figure 1 visualises caseload and hospital numbers of both data sources and depicts that our study population lack 3.1 to 4.9% of cases, and 2.3 to 12.3% of hospitals per year (Table 4 contains all data in detail).

**Table 5:** Study population: annual distribution of hospitals with and without total knee arthroplasty (TKA), complying with minimum volume requirements, and case numbers

	1055 hospitals of the study population (100%)			Hospitals < 50 TKA per year		Hospitals ≥ 50 TKA per year	
	Hospitals (%) with TKA	Cases treated in hospitals with TKA (100%)	Hospitals (%) without TKA	Hospitals (%)	Cases (%)	Hospitals (%)	Cases (%)
**2006**	888 (84.2%)	120,700	167 (15.8%)	129 (12.2%)	3489 (2.9%)	759 (71.9%)	117,211 (97.1%)
**2008**	926 (87.8%)	141,714	129 (12.2%)	96 (9.1%)	2664 (1.9%)	830 (78.7%)	139,050 (98.1%)
**2010**	956 (90.6%)	140,866	99 (9.4%)	103 (9.8%)	2837 (2.0%)	853 (80.9%)	138,029 (98.0%)
**2012**	987 (93.6%)	127,404	68 (6.5%)	206 (19.5%)	5837 (4.6%)	781 (74.0%)	121,567 (95.2%)
**2013**	1007 (95.5%)	121,134	48 (4.6%)	270 (25.6%)	7643 (6.3%)	737 (69.9%)	113,491 (93.7%)
**2014**	1017 (96.4%)	124,544	38 (3.6%)	262 (24.8%)	7095 (5.7%)	755 (71.6%)	117,449 (94.3%)

The number of hospitals performing less than 50 cases per year doubled from 103 (9.8%) in 2010 with TKA-MVRQ to 206 (19.5%) in 2012 and reached some 25% in 2013 and 2014 without TKA-MVRQ. Their cases doubled as well from 2837 in 2010 to 5837 in 2012, amounting to 4.6% of all cases, and rising to 6% in the 2 years following. In 2014, an additional 159 hospitals performed less than 50 cases as compared to 2010. This increase can be attributed both to the initial 98 hospitals meeting TKA-MVRQ and to 61 hospitals not previously performing TKA.

The multifactorial analysis of variance with repeated measures shows that only time significantly influences (*p* < 0.001) whether hospitals continued performing 50 or more TKA annually or less without MVRQ, while hospital size, geographical region, type of ownership, and interactions of time with the latter three variables show no significant effects (Table 6). Post-hoc-tests reveal a significant decrease of suspending the MVRQ in 2012. The average number of hospitals performing at least 50 TKA declined from 88.1–91.6% before to a level of 77.9–73.3% afterwards (Table 7). Table 8 displays the corresponding results of the Friedman test with pairwise comparison of case numbers in the 6 years under observation, the significance values at hand are Bonferroni-corrected.

**Table 6:** Multifactorial analysis of variance with repeated measures on hospitals performing less than 50 TKA cases or more per year, Greenhouse–Geisser-adjusted

	df	F	Sig	Partial eta squared
**Type of ownership**	2	0.347	.707	.001
**Hospital size**	4	.870	.046	.010
**Region**	2	.373	.354	.002
**Time**	5	33,975	<.001	.033
Time*hospital size	15.66	.584	.896	.002
Time* region	8.20	1.086	.370	.002
Time*type of ownership	7.83	.911	.504	.002

**Table 7:** Changes of Hospital Case numbers of TKA over time with Post-Hoc-Tests

	Percentage of hospitals with caseload ≥ 50/year				Post-hoc-tests (Bonferroni-correction)					
	Mean	SD	95%-confidence interval							
			Lower limit	Upper limit	2006	2008	2010	2012	2013	2014
**2006**	88.06	1.68	84.77	91.35	x					
**2008**	91.63	1.47	88.74	94.53	0.916	x				
**2010**	90.13	1.51	87.17	93.10	1.000	1.000	x			
**2012**	77.94	2.01	74.0	81.87	< 0.001	< 0.001	< 0.001	x		
**2013**	71.59	2.23	67.21	75.96	< 0.001	< 0.001	< 0.001	0.011	x	
**2014**	73.27	2.2	68.95	77.60	< 0.001	< 0.001	< 0.001	0.407	1.000	x

**Table 8:** **Friedman test (nonparametric) of Hospital Case numbers of TKA over time with Post-Hoc-Tests**

	Friedman test					Post-hoc-tests (Dunn-Bonferroni-Test) with Bonferroni-correction					
	N	Mean	SD	Mean rank	Sig.	2006	2008	2010	2012	2013	2014
**2006**	1055	114.41	125,58	3,10	0,000	x					
**2008**	1055	134,33	139,28	4,08		< 0.001	x				
**2010**	1055	133,52	135,30	4,10		< 0.001	1.000	x			
**2012**	1055	120,76	123,68	3,48		< 0.001	< 0.001	< 0.001	x		
**2013**	1055	114,82	123,31	3,00		1.000	< 0.001	< 0.001	< 0.001	x	
**2014**	1055	118,05	128,60	3,24		1.000	< 0.001	< 0.001	.037	.050	x

### Study aim two

Mann-Whitney-U-tests reveal that three out of four evaluable TKA-QIs show significantly higher quality values in hospitals with < 50 cases in all years. Only the QI ‘postoperative mobility of at least 0/0/90 degrees’ shows no significant difference in this respect in any year (Table 9).

**Table 9:** Comparison of quality indicators between hospitals with a caseload ≥50 vs. < 50 of total knee arthroplasty (TKA) per year

		2010		2012		2013		2014	
**TKA Quality Indicator**	**Hospital case load**	**Hospital n**	**Mean rank**	**Hospital n**	**Mean rank**	**Hospital n**	**Mean rank**	**Hospital n**	**Mean rank**
**TKA meeting indication criteria**	< 50 cases	94	542.3	195	549.6	251	516.8	241	539.1
	≥50 cases	823	449.5	762	460.9	723	477.3	749	481.5
	p		0.001		< 0.001		0.052		0.005
**Perioperative antibiotic-prophylaxis applied**	< 50 cases			198	548.2	251	557.0	244	560.0
	≥50 cases			764	464.2	724	464.1	751	477.9
	p				< 0.001		< 0.001		< 0.001
**Postoperative mobility (neutral zero method) measured**	< 50 cases	92	516.6	194	570.2	251	587.8	240	545.1
	≥50 cases	823	451.5	763	455.8	723	452.7	750	479.6
	p		0.016		< 0.001		< 0.001		0.001
**Postoperative mobility of at least 0/0/90 (neutral zero method)**	< 50 cases	91	435.7	194	493.6	245	483.1	233	504.2
	≥50 cases	820	458.3	761	474.0	724	485.7	749	487.6
	p		0.438		0.377		0.899		0.434

The effect of TKA-MVRQ suspension on TKA-QI values, tested with a multifactorial MANOVA with a paired sample, shows significant quality differences for all four QIs (Table 10). The post-hoc-tests show a significant improvement for all four QIs between the first and second year of public indicator reporting, thus coinciding with the intervention timeline of 2010 to 2012 (Table 10), as well as a continuous significant difference between all 4 years for postoperative mobility measured by the neutral zero method, indicating a steady improvement.

**Table 10:** Effect of minimum volume requirements suspension in 2011 on quality indicator values for total knee arthroplasty (TKA)

	Multifactorial MANOVA, paired sample					Bonferroni-adjusted post-hoc-tests		
**TKA Quality Indicator**		**2010**	**2012**	**2013**	**2014**	**2010 versus 2012**	**2012 versus 2013**	**2013 versus 2014**
**TKA meets indication criteria**	Mean	94.78	95.91	96.06	96.57			
	p	< 0.001				< 0.001	1.000	0.223
**Perioperative antibiotic-prophylaxis applied**	Mean		99.62	99.77	99.66			
	p	< 0.001					0.007	0.295
**Postoperative mobility (neutral zero method) measured**	Mean	97.32	98.37	97.68	98.46			
	p	< 0.001				0.003	0.002	< 0.001
**Postoperative mobility of at least 0/0/90 (neutral zero method)**	Mean	88.22	90.84	91.59	91.71			
	p	< 0.001				< 0.001	0.160	1.000

## Discussion

The minimum volume requirement of 50 TKA cases per year does affect hospitals’ performance in Germany. Our study results show the effect of MVRQs in force for the first time by studying the effect of suspension and reintroduction on an entire health care system, taking the methodological limitations of a before-after study design with an uncontrolled and unplanned intervention into account. Hospitals in Germany increased their TKA frequency with MVRQ in force and reduced them with suspended MVRQ while the number of performing hospitals remained near-constant. Our study results on MVRQ’s effect on quality is restricted due to data availability.

TKA cases already rose by a third from 110,349 in 2004, when TKA-MVRQs were first officially announced, and before their introduction in 2006, to some 146,747 cases in 2010 afterwards. Meanwhile, the number of performing hospitals remained near-constant at about 1030. The salient rise of about 130 hospitals since 2014 is due to a preciser reporting in the obligatory case documentation for external quality assurance. Hospitals in a hospital network have to identify the discharging hospital site where a patient received TKA. In addition, the Federal Joint Committee introduced a positive list of hospital sites expected to report as well as monetary sanctions for noncompliance to enforce site-specific reporting in the quality reports. These changes lead to a higher number of hospitals in statistical reporting, while the de facto number of hospitals performing TKA remained constant. Thus, the increase in TKA numbers indicate a steering effect of MRVQ, while the constant de facto number of performing hospitals may correspond to the finding that no regionalisation effect was observed under MVRG in Germany until 2010 [20].

The steering effect of TKA-MVRQ was reversed unintentionally by Federal Joint Committee’s decision to suspend TKA-MVRQ from 2011 onwards, induced by a legal dispute on the freedom of medical treatment and the evidence for the respective threshold of 50 cases per year. Its confirming verdict in autumn 2014 allowed the Federal Joint Committee to reintroduce the unchanged TKA-MVRQ in 2015. This time, the caseload fell by 11%, or about 17,000 to around 130,000 annual cases after suspension, just to rise once more following its reintroduction by 13% or about 17,000 cases to 148,000 in 2017, back to its high in 2009.

Wengler et al. compared the rise of TKA cases in Germany between 2005 and 2011 to the corresponding increase of TKA cases in the USA, finding that 8.3 percentage points of the 21.6% increment in Germany were due to demographic changes, and 12.3 percentage points due to non-demographic reasons [51]. According to a recent review by Price et al. on TKA, these non-demographic reasons which contribute to variation in the use of knee arthroplasty surgery are an interplay of economic variables, health-care system factors, reimbursement, and patient and surgeon preferences [52]. Questioning the rise and fall of TKA cases in Germany, the rise during its first introduction in 2006 coincided with the last phase of a systemwide change of hospital reimbursement from daily nursing charges to diagnosis-related groups, known to induce rising case numbers. Most likely, the rise was a result of an interplay of a health care system factor, namely the introduction of MVRQ for TKA, and a change in reimbursement. For the period 2011 to 2015 the main effect can be attributed to the health-care system factor, consisting in the unplanned and unintended intervention of the Federal Joint Committee to intermit the MVRQ for TKA. During this period, neither sudden changes in the epidemiology of diseases leading to TKA, nor demographic changes, nor changes in the technical operation procedure, the diagnostic process, indication, or the remuneration occurred. Thus, these factors had no impact on the reduction of TKA cases from 2011 to 2013, or on the rise from 2014 to 2017. But it is to note that from 2011 to 2012, the documentation procedure for the case identification of TKA in the external quality assurance programme was changed. According to the external quality assurance annual report in 2012 [53], this explains 4% points of the 8% case reduction in the year 2012. But it does not explain either the further reduction in 2013 or the following rise from 2014 on.

But the hospital-based data of the study population present more detailed information on the steering effect of the TKA-MVRQ on hospitals than federally aggregated external quality assurance data. After TKA-MVRQ suspension, the number of hospitals performing TKA below 50 cases rose from 10 to 25%. And it is most important to keep in mind that our analyses revealed no influence of hospital size, its type of ownership or the region within Germany on the adaption to the MVRQ. It is sheer time, i.e. the MVRQ, which changes the hospitals’ performance volume. Looking at the patient, the data on hospital level show that, with TKA-MVRQ in force, 2% or about 2800 patients were treated in hospitals not meeting the annual caseload of 50. This number rose to more than 7000 patients, i.e. approx. 6% of all cases. Regarding overall treatment quality for patients, reviewing the originally intended steering effect of MVRQs is most important. It is supposed to avoid the treatment of patients in hospitals with very small caseloads and, instead, ensure the chance of better treatment quality by transferring the patients to hospitals with higher caseloads. The data implies two steering effects. The intended inter-hospital steering effect is smaller and can be seen in those 61 hospitals that begin performing TKA once the TKA-MVRQ was suspended, and remain under 50 cases instead of referring TKA patients to other hospitals directly (or indirectly by not offering the procedure). They account for 5.8% of all TKA-performing hospitals of the study population. The intra-hospital caseload adoption practised by 9.5% of all hospitals indicates a second steering effect. Almost 100 of the 853 TKA-performing hospitals meeting the MVRQ before suspension reduced their caseload below 50 per year once the TKA-MVRQ was suspended.

The data suggest that hospitals do react to MVRQs but their main focus of action is confined to their hospital. However, for the sake of the patient, an inter-hospital perspective including regional cooperation with other hospitals is still to be fostered by health policy. From the hospital’s perspective, it might be difficult to be economically successful in light of the DRG reimbursement system and, at the same time, cooperate with other hospitals and thus forego a number of cases. To encourage regionalisation, health policymakers could increase the minimum volume threshold considerably, thus making it more difficult (and less probable) for hospitals to extend their caseloads successfully. Another, more complex option would be the promotion of regional quality cooperation as done in the Netherlands, integrating all health care providers [54, 55].

MVRQs are introduced in order to ameliorate treatment quality. International studies demonstrate better results on mortality, complications, and required reoperations for TKA as outlined in the introduction. So far, in Germany, only Mansky’s working group evaluated the quality of hospital procedures underlying MVRQs on a nationwide scale. They found lower in-hospital mortality for TKA with higher case volumes [21]. Their data show 843 deaths among 842,844 TKA cases for the 6 years from 2009 up to 2014, resulting in a mortality rate of 0.10% varying between 0.13% for very low volume hospitals with a median annual volume of 56 cases and 0.06% for very high-volume hospitals with a median annual volume of 292 cases [25]. These data correspond impressively with the aggregated external quality assurance data of the same 6 years counting 823 deaths among 830,548 cases yielding the same mean mortality rate of 0.10% showing a reduction by more than 50% since the 1980s [39, 40] and indicating that mortality is a rare event in TKA and confirming that a broader set of quality indicators should be applied. Our study evaluates the quality analysing those TKA-QI results of the external quality assurance programme reported publicly on the hospital level. These data are limited. Six out of ten QIs could not be used due to data privacy restrictions when reporting small numbers. Therefore, no data on the outcome QI ‘in-hospital mortality’, ‘reoperation’, and ‘postoperative wound infection’ could be analysed and hence cannot be compared with the cited literature. The only outcome QI, i.e. ‘achieving a postoperative mobility of at least 0/0/90’, does not differ significantly. Surprisingly, the results for three out of four evaluable indication- and process-related QIs are counter-intuitive: Hospitals with less than 50 cases show better results in meeting indication criteria, administering perioperative antibiotic-prophylaxis, and assessing postoperative mobility by the neutral zero method than those with more than 50 cases. Interpreting these results, one has to consider that (1) all QIs are not case-adjusted, (2) the absolute quality value differences are small, and (3) a ceiling effect is present; thus, statistically significant differences might not correspond to clinical differences.

The answer to the question, whether MVRQs affect quality, remains ambivalent. In the longitudinal analysis, all four indicators show a significant quality improvement that can be linked to the year following the initial compulsory public reporting, and three do not show significant improvement afterwards. Only the QI ‘postoperative mobility measurement’ continues to rise significantly. It is a phenomenon observed before [24, 56], questioning the interplay of public reporting and quality improvement. Nevertheless, three QI improve in 2012, i.e. coincident with the first year after suspending the TKA-MVRQ.

All in all, our findings on quality have to be interpreted cautiously. The majority of outcome indicators are not available for TKA evaluation due to low incidences, and guideline-based process indicators show ceiling effects. It might be necessary to broaden the quality dimensions monitored, since almost one out of five patients is dissatisfied after TKA and experiences ongoing pain and poor function [52]. It might be helpful to consider patient-reported outcomes and a longer time frame alongside the mentioned and more short-term oriented QIs to gain a comprehensive and complete picture of TKA quality.

## Conclusions

The minimum volume requirements (MVRQs) for TKA indicate a steering effect on Germany’s hospitals: introducing TKA-MVRQ was associated with an increase in caseloads, suspending TKA-MVRQ with a decrease, and reintroducing TKA-MVRQ replicated the increase. The intra-hospital effect appears to prevail over the intended inter-hospital case relocation regarding caseload adaption. Introducing MVRQs seems to increase the frequency of TKA. Health policy should study and reflect not only single measures and their steering effects but consider the conjunction of diverse measures introduced by regulations, and learn about their dynamic interactions.

## Data Availability

All data used are publicly available: The Federal Joint Committee (Gemeinsamer Bundesausschuss, G-BA) provides all hospital quality records on request (https://www.g-ba.de/themen/qualitaetssicherung/datenerhebung-zur-qualitaetssicherung/datenerhebung-qualitaetsbericht/); the Institute for Quality Assurance and Transparency in Healthcare (IQTiG) provides the aggregated data of the German external hospital quality assurance programme on its website (https://iqtig.org/index/).

## Abbreviations

MANOVA: Multivariate analysis of variance
MVRG: Minimum volume regulation
MVRQs: Minimum volume requirements
QI: Quality indicator
TKA: Total knee arthroplasty
TKA-MVRQ: Minimum volume requirements on total knee arthroplasty
TKA-QIs: Total knee arthroplasty quality indicators
